# Are *Colpodella* Species Pathogenic? Nutrient Uptake and Approaches to Diagnose Infections

**DOI:** 10.3390/pathogens13070600

**Published:** 2024-07-21

**Authors:** Mahdi I. Salti, Tobili Y. Sam-Yellowe

**Affiliations:** Department of Biological, Geological and Environmental Sciences, Cleveland State University, Cleveland, OH 44115, USA; m.i.salti@vikes.csuohio.edu

**Keywords:** apicomplexa, *Colpodella* species, *Colpodella* sp. ATCC 50594, *Colpodella* infection, Sam-Yellowe’s trichrome staining, endocytosis, myzocytosis, phagotropy, trogocytosis

## Abstract

*Colpodella* species are free-living protists phylogenetically related to apicomplexans. *Colpodella* sp. have been detected in human and animal tissues, as well as in ticks and biting flies. The trophozoite and cyst stages of *Colpodella* species can be distinguished from stages of the prey *Parabodo caudatus* using Sam-Yellowe’s trichrome staining. *Colpodella* species obtain nutrients by attaching to their prey, aspirating the prey’s cytoplasmic contents into a posterior food vacuole and encysting. It is unclear whether both trophozoite and cyst stages are present in human and animal tissues. Molecular techniques have detected *Colpodella* species in human blood, cerebrospinal fluid, and in ticks and flies. However, no morphological information was reported to aid life-cycle stage identification of *Colpodella* species. This review discusses the increased reports of *Colpodella* species detection in animals and in arthropods and the need to identify stages present in human and animal tissues. We previously used Sam-Yellowe’s trichrome staining to identify life-cycle stages of *Colpodella* sp. In this review, we examine the reports of *Colpodella* species detection in human and animal tissues to determine whether the identification of *Colpodella* species represents true infections or contaminations of samples collected during routine surveillance of piroplasm infections in animals and arthropods. This review also aims to provide insights regarding *Colpodella*, nutrient uptake, and the survival of *Colpodella* sp. within humans, animals, and arthropods, as well as whether the attachment of trophozoites to cells occurs in tissues leading to myzocytosis and endocytosis.

## 1. Introduction

*Colpodella* species are free-living predatory protists classified as myzozoans, a diverse group of organisms that includes dinoflagellates, apicomplexans, and predatory biflagellated free-living protists. They possess an apical complex consisting of secretory organelles like the rhoptries and micronemes [1,2]. A pseudoconoid (open conoid) contained within the rostrum is used for attachment and predation among the free-living protists, in a process known as myzocytosis where cytoplasmic contents of the prey are aspirated into the predator. In *Plasmodium* species, *Toxoplasma gondii, Cryptosporidium* spp., and among the gregarines, apical complex organelles also participate in nutrient uptake along with invasion [2,3,4]. The feeding of gregarines, *Colpodella tetrahymenae*, and *Colpodella gonderi* is characterized as extracellular parasitism, while *Cryptosporidium* spp. forms a feeder organelle as an epicellular parasite of epithelial cells in the gastrointestinal tract [3,5,6,7]. Feeding on biflagellated bodonid species and algae using the process of myzocytosis leads to the formation of a cyst in some *Colpodella* species. Non-cyst forming species such as *Colpodella unguis* and *Colpodella edax* have been described [8,9]. Free-living *Colpodella* species have been reported to cause infections in humans and in animals [10,11,12,13,14,15]. *Colpodella* sp. have also been detected in ticks and biting flies [11,16], prompting the concern for transmission to humans and animals through tick and fly bites.

## 2. Detection of *Colpodella* Species in Humans and Animals

A babesiosis-like relapsing fever, with red blood cell infection was reported in a 57-year-old woman in Yunnan Province, China [10]. A polymerase chain reaction targeting the 18S rRNA gene and sequence analysis showed that the identified *Colpodella* sp. had an 89% similarity with *Colpodella tetrahymenae* [10]. A second human case of *Colpodella* infection was reported in a 55-year-old woman in Heilongjiang Province, in northeast China [11]. Neurological symptoms developed following a tick bite. Four hundred and seventy-four *Ixodes persulcatus* ticks were examined for *Colpodella* sp. from the woodlands surrounding her residence. Two ticks were positive for *Colpodella* [10,11]. In both human cases, the identification of the transmission and pathogenic stages of *Colpodella* were not performed (NP), reported, or confirmed by light or electron microscopy (Table 1). *C. gonderi* was identified in a human case of urinary tract infection, although the cause of infection was uncertain [17]. Giemsa staining for light microscopy was performed with the identification of trophozoite stages in urine. However, transmission or pathogenic stages were not described. It was also unclear if C. *gonderi* was the etiological agent for the infection [17].

**Figure 1:**
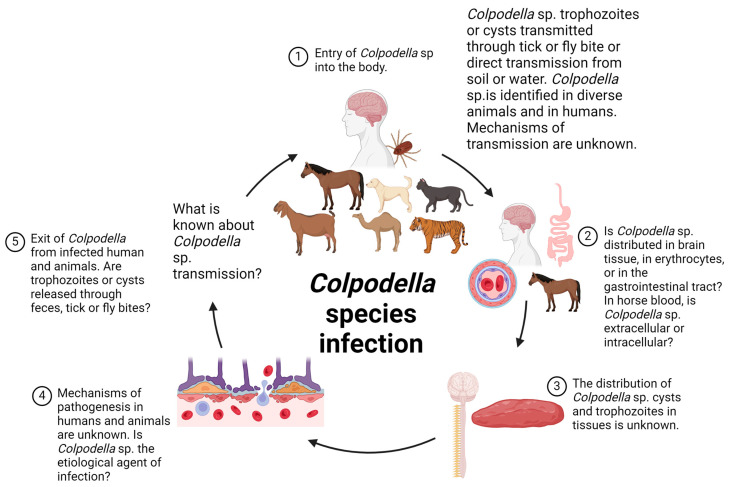
Proposed life cycle of *Colpodella* sp. infection in humans, animals, and arthropods. The mechanisms of transmission are unknown. *Colpodella* species were identified in diverse animals such as horses, camels, dogs, goats, and domestic and wild cats but are mainly associated with ticks. Distribution in tissues is unknown, nutrients needed for survival are unknown, and the mechanism of tissue damage is unknown. Whether *Colpodella* sp. detection in humans and animals represents true infections similar to infections caused by a pathogenic apicomplexan or not is unknown. It is unclear if *Colpodella* sp. obtain nutrients by endocytosis or myzocytosis in human and animal tissues. The figure was created using BioRender.com (accessed on 29 June 2024).

*Colpodella* sp. have been detected in diverse animals including cattle, ticks infesting cattle, raccoons (*Procyon lotor*), horses, in fecal samples from zoo felines, domestic (pet) cats and dogs, and in ticks attached to goats [18,19,20,21,22] (Table 1, Figure 1). A routine screening of whole blood samples from 400 horses identified *Colpodella* species in two samples, along with *Babesia caballi* in two samples and *Theileria equi* in 132 samples [18]. However, the tissue location and distribution in these animals is unknown [18,19,20,21,22]. These observations suggest that ticks may be potential vectors for transmission and that *Colpodella* species may cause opportunistic infection [18,19,20,21,22]. Alternately, *Colpodella* sp. may be contaminants from the soil or water. They may be transported in ticks and flies mechanically, without biological development, or they may undergo development and differentiation of life-cycle stages with the ticks serving as biological vectors. We currently lack information regarding how transmission can occur, and further investigations are needed to provide insights into the infectivity of *Colpodella* species and the presence of virulence factors that could aid pathogenicity. *Colpodella* species have been identified in a tick causing infection in a human host and in ticks infesting animals [11,19,22]. Tabanid flies and *Stomoxys indicus* were shown to be positive for *Colpodella* species on horse farms in Thailand [16]. Three different *Colpodella* species were identified in raccoons (*P. lotor*), with the suggestion that raccoons may serve as “dispersal vectors” for *Colpodella* sp. [20]. *Colpodella* 18S rDNA was identified from the blood of a South China Tiger (*Panthera tigris amoyensis* Hilheimer) that died of infection from a tick bite [12]. The DNA sequence from the identified *Colpodella* sp. had a 90.1% sequence identity to *Colpodella* species strain HEP [10,12] and a 90.4% similarity to *Colpodella* sp. strain Heilonjiang (HLJ) [11,12] (Table 1). Chiu et al. [12], reported symptoms of severe jaundice and enlarged organs in the babesiosis-like infection in the South China Tiger. Although PCR and DNA sequencing identified *Colpodella* sp., it is unclear if *Colpodella* sp. was the etiological agent for the infection. Out of 402 adult ticks examined from the tiger enclosure and grasses around the enclosure, 22 were positive for *Colpodella* species [12]. Two *Colpodella* species with sequence homology to *Colpodella* sp. (ATCC 50594) were identified in horse blood [18]. None of the animal studies reported life-cycle stage identification through staining for light microscopy, differential interference contrast (DIC) microscopy, or by electron microscopy. The morphology of the *Colpodella* species identified is unknown. The life-cycle stages involved in transmission are unknown, and the mechanism of infection, including the types of nutrients taken up from the host during infection, have not been described. It is unclear how *Colpodella* sp. survive in mammalian species and in arthropods.

## 3. Patterns of Nutrient Uptake in *Colpodella* Species

Among myzozoans, predators aspirate large particulate material from the prey’s cytoplasm during myzocytosis. This suggests that nutrient uptake occurs using the pseudoconoid and serves as an early mode of attachment to prey and is similar to nutrient uptake in the basal apicomplexan lineages such as in archigregarines and in free-living myzozoans [3]. The position and function of the food vacuole among apicomplexans like *Selenidium pendula* Giard, 1884, which feeds by myzocytosis, may influence the development of the life-cycle stages following nutrient uptake. A flask-shaped organelle found to contain digested pieces of host cell organelles and debris was thought to be the food vacuole, with nutrient uptake resulting from phagocytosis in *S. pendula* Giard, 1884 [26]. The presence of a posterior food vacuole in *Colpodella* sp. (ATCC 50594) differs from the food vacuoles located in the anterior end of the trophozoite in *S. pendula* Giard, 1884 [26] and is located near rhoptries in the anterior end. Trophozoites of *Colpodella* sp. (ATCC 50594) initiate myzocytosis by binding with a myzocytic aperture posterior to the apical tip and can feed intermittently on multiple prey. *Colpodella* species can attach to prey, commence feeding, and then detach from the prey to seek new prey [27]. In previous studies, we showed that the process of myzocytosis in *Colpodella* sp. (ATCC 50594) occurs sequentially, beginning with attachment to the prey *Parabodo caudatus*, engulfment of the plasma membrane of the prey, destruction of the prey’s plasma membrane, and aspiration of the prey’s cytoplasmic contents into a posterior food vacuole [28]. In addition to myzocytosis for nutrient uptake, *Colpodella* sp. (ATCC 50594) trophozoites can also carry out endocytosis in culture [28]. Following myzocytosis and nutrient acquisition, *Colpodella* sp. (ATCC 50594) encysts. Nutrients transported to the posterior food vacuole aid cyst development and maturation. This is followed by mitosis and cytokinesis to produce juvenile trophozoites. Different methods are used by pathogenic protists to obtain nutrients within their hosts. *Cryptosporidium* spp. use a feeder organelle that is formed at the parasite–host cell interface for obtaining nutrients from the host cell [29]. Free-living opportunistic amoeba such as *Naegleria fowleri* that infect the nervous system of the host through the olfactory nerve use food cups (amoebastomes), which are cytoplasmic extensions of the amoeba surface, to digest brain tissue [30]. The biflagellate trophozoite stage of *N. fowleri* initiates infection in human and animal infections. Target cells in the brain are destroyed by “nibbling” and ingestion of the host tissue in a process known as trogocytosis. The process of adhesion and attachment have been identified by microcopy with *Naegleria fowleri*-actin (Nf-actin) identified by immunofluorescence [31,32]. Both cyst and trophozoite stages of *N. fowleri* were identified in infected host specimens using light microscopy [33]. Investigations aimed at providing an accurate diagnosis of amoebic keratitis to distinguish *Acanthamoeba* from non-*Acanthamoeba* amoebic keratitis and the presence of mixed infections show the importance of using a combination of methods to identify and accurately diagnose infection. The use of culture, microscopy, and PCR is emphasized for identifying parasite stages [34]. Cytolytic effects of *Acanthamoeba castellanii* in vitro have been identified by light microscopy [35] and the phagocytosis of erythrocytes, leukocytes, and bacteria by *Trichomonas vaginalis* in vitro demonstrates how *T. vaginalis* obtains nutrients during host infection [36].

## 4. Are *Colpodella* Species True Pathogens?

Are the reports of *Colpodella* sp. in humans, animals, and arthropods true infections, infestations, or a contamination of specimens from the soil and aquatic environments? If these are indeed true opportunistic infections leading to pathogenesis, then the morphological identity of *Colpodella* sp. life-cycle stages is urgently needed to aid the better characterization and prioritization of which species and strains of *Colpodella* to emphasize in investigations. *Colpodella* sp. can be cultured in vitro in diprotist cultures using a Hay medium, allowing for investigations in vitro [27]. Giemsa staining is routinely used to stain specimens containing parasitic protists. However, the differentiation of life-cycle stages may be challenging depending on the life-cycle stage that needs to be identified. We developed Sam-Yellowe’s trichrome staining protocols to identify life-cycle stages of *Colpodella* sp. (ATCC 50594) [37]. The staining protocol clearly distinguishes cyst stages of predator and prey. Developmental stages of cysts and trophozoites can be identified [37]. The staining protocol was used to identify previously undocumented life-cycle stages of *Colpodella* sp. (ATCC 50594) and facilitated interpretations of transmission electron micrographs [27,38]. Staining specimens obtained from *Colpodella*-infected hosts and from the arthropod vectors for light microscopy will provide a better understanding of *Colpodella* interaction with human and animal tissues, identify life-cycle stages present in the tissues, and determine stages involved in transmission and pathogenesis. We showed in previous studies that unattached *Colpodella* sp. (ATCC 50594) trophozoites can endocytose nanoparticles of 40 and 100 nm from culture, suggesting that, in addition to myzocytosis, the predator can acquire nutrients by endocytosis [28]. Whether endocytosis is sufficient to form the food vacuole and lead to encystation is unknown and requires further investigation. The process of endocytosis may be used by *Colpodella* species for nutrient uptake and survival in human, animal, and arthropod tissues. Alternately, *Colpodella* sp. may carry out contact-dependent interaction with cells, leading to cell and tissue destruction or invade human cells as described [10]. Understanding the biology of *Colpodella* species is crucial to identifying transmission stages initiating infection in humans and animals and identifying stages of *Colpodella* associated with pathogenesis. Morphological identification of life-cycle stages by staining and light microscopy is required to identify the distribution of the protist within infected host tissues. Markers identifying transmission and pathogenic stages of *Colpodella* sp. in the life cycle are unknown. In *Plasmodium falciparum*-infected erythrocytes, uptake of the host cytosol into the food vacuole has been described using fluorescent dextran, which was identified in vesicles inside the intracellular parasite [39]. Proteins having roles in endocytosis such as Kelch 13, AP-2µ, and Eps-15 were identified in *P. falciparum* as markers of endocytosis [39,40,41]. Additionally, the protein VPS45 identified in *P. falciparum* is involved in host-cell cytosol uptake (HCCU) [39]. Inactivation of the genes encoding these proteins resulted in decreased hemoglobin uptake [39,40,41,42]. In future experiments, it will be important to identify markers for endocytosis, myzocytosis, encystation, and excystation in *Colpodella* species. In particular, Kelch 13, a protein associated with endocytosis, has been identified in all apicomplexans and myzozoans examined [43], and its presence and role in endocytosis will provide key insights into the similarities of endocytosis across the apicomplexa, including in *Colpodella* species detected in human and animal tissues. Apicomplexans utilize apical phagotrophy, phagocytosis, osmotrophy, pinocytosis, and endocytosis for nutrient uptake, with the cytostome and micropore implicated for endocytosis [3,29].

## 5. Myzocytosis, Endocytosis, and Role of the Food Vacuole in Nutrient Uptake

Brugerolle [44] described the ultrastructure of *Colpodella vorax*, showing aspiration of the prey’s organelles though a channel formed after attachment and the resultant encystation following feeding. Similarly, *C. tetrahymenae*, ectoparasitic to the ciliate *Tetrahymena* aff. *pyriformis*, encysts following myzocytosis. However, an enlargement of the food vacuole and a precyst stage was not described [6]. The use of staining protocols such as Giemsa and Sam-Yellowe’s trichrome staining, which can be performed in less than 10 min, will provide important insights regarding morphological similarities and differences in each of the infections described and in the ticks and flies shown to harbor *Colpodella* species. Two new species of *Colpodella*, *Colpodella* sp. *struthionis* and *Colpodella* sp. *yiyuansis*, were named by Qi et al. [22]. However, morphological characteristics of the cells were not described, the infectivity of the life-cycle stages are unknown, the mode of survival and nutrient uptake within the infected hosts are unknown, and the presence of food vacuoles is unknown. Endocytosis has not been described in other *Colpodella* species besides *Colpodella* sp. (ATCC 50594). Therefore, it is unclear whether similar mechanisms are used, particularly in species that feed on ciliates and algae. The biology of *Colpodella* species is still unclear. Although investigations of the model *Colpodella* sp. (ATCC 50594) is beginning to provide insights into life-cycle stage transitions, the diprotist culture conditions in bacterized media pose a challenge to studies focused on *Colpodella* sp. (ATCC 50594). This is due to the presence of *Colpodella* sp. (ATCC 50594), which preys on the bacteriotrophic *P. caudatus* in the same culture. The bacteria in the culture serve as food for *P. caudatus*. If the presence of *Colpodella* sp. in human and animal tissues constitutes true infections, the effects of infection on human and animal tissues will need to be investigated in order to understand the mechanisms of pathogenesis, as well as tissue specificity and the tropism of life-cycle stages once inside the human and animal tissues. Investigators reporting the detection of *Colpodella* species in animals and arthropods consider this an area of concern due to potential tick-borne infections in humans [18,45]. The identification of *Colpodella* species in a wide range of animals such as cattle [23], horses [18], and raccoons [20] and in the ticks infesting animals such as the ticks infesting camels [24] and goats [22], poses a public health concern due to potential tick bites in humans in close contact with these animals. So far, *Colpodella* species have been identified in the ticks *Ixodes persulcatus*, *Rhipicephalus microplus, Rhipicephalus bursa, Dermacentor*, *Haemaphysalis longicornis*, and *Hyalomma dromedarii* [11,14,15,19,22,24,25]. These ticks are found to infest animals that are constantly in close contact with humans, such as work animals, agricultural animals, pets, and recreational animals associated with tourists [11,14,15,19,22,24,25]. The presence of *Colpodella* species in humans, animals, and arthropods and the mechanisms of transmission and pathogenicity merit further investigation. The reports of pathogenesis in the two human cases where *Colpodella* species was detected and in the tissue damage and pathology described in a domestic and wild cat did not identify *Colpodella* sp. life-cycle stages in the tissues [12,13].

## 6. Understanding the Mechanism of Transmission and Survival within Humans and Animals

How *Colpodella* species survive within the body and what nutrient sources promote cell survival is currently unknown. The following questions will need to be answered to provide clarity about the mechanisms of transmission and pathogenesis of *Colpodella* sp. putative infections. (1) What are the life-cycle stages of *Colpodella* species causing infection and pathogenesis? (2) Are *Colpodella* trophozoites able to attach to and feed on host cells such as erythrocytes, leukocytes, or epithelial cells? (3) Can *Colpodella* sp. trophozoites invade host cells? (4) Myzocytosis in culture and in the environment occurs when the *Colplodella* sp. trophozoite engulfs the plasma membrane of the prey, dissolves the membrane, and aspirates the cytoplasmic contents of the prey. What is the nutrient source for *Colpodella* sp. in human and animal tissues? (5) Where in the ticks and flies are *Colpodella* sp. life-cycle stages located? (6) What is the nutrient source for *Colpodella* within the ticks and flies? (7) Are ticks and flies merely transporting *Colpodella* sp. as mechanical vectors, or does development and differentiation occur to produce infective *Colpodella* sp. stages, with the arthropods serving as biological vectors? (8) Is a food vacuole formed, and are cyst stages formed within the host? (9) Do *Colpodella* sp. coinfections occur with apicomplexans such as *Plasmodium* species, *Toxoplasma gondii, Babesia* species, or *Theilaria* species? The use of culture and microscopy along with molecular methods is necessary for the identification of the life-cycle stages transmitting infection and causing pathogenesis [34]. Giemsa staining has been useful in identifying trophozoites of *Colpodella* sp. and its prey *P. caudatus*, particularly being able to differentiate the kinetoplast and nucleus of the prey. Sam-Yellowe’s trichrome staining can identify and differentiate precyst and cyst stages of the predator and prey and help with the identification of the stage of maturity of both trophozoites and cysts if present in tissue specimens. Additional investigations will be needed to identify markers of transmission and pathogenesis. It will be important to know if recently identified species are different from previously described species. A morphological identification of life-cycle stages of *Colpodella* sp. obtained from humans, animals, and arthropods, stained for light microscopy, will provide much-needed information regarding the morphology of transmission stages and the distribution of life-cycle stages present in the tissues. Diagnosis using molecular techniques, while very useful, should be aided by staining for light microscopy, further evaluation of the ultrastructure of the identified *Colpodella* species, and culturing the cells to allow for further cell biological and molecular investigations required to aid clinical investigations and diagnosis.

## Figures and Tables

**Table 1:** *Colpodella* species detected in humans, animals, ticks and flies.

	Research Study Reference	Year of Publication	Location	Host Species	Tick/Flies Species	Staining for Light Microscopy	Identification Method	DNA Sequence Homology with *Colpodella* sp.
1	[10]	2012	Kunming City, Yunnan Province, China	Human	N/A	Giemsa Stain	Polymerase Chain Reaction	*Colpodella tetrahymenae* (89% similarity)
2	[11]	2018	Heilongjiang Province, China	Human	*Ixodes persulcatus*	NP	Polymerase Chain Reaction	*Colpodella* sp. (89–90% similarity)
3	[12]	2022	Meihua Mountains, Fujian, China	Tiger	Unidentified Tick	NP	Polymerase Chain Reaction	*Colpodella* sp. (91.1% similarity to *Colpodella* sp. strain human erythrocyte parasite (HEP, MH208621) and 90.4% similar to the *Colpodella* sp. strain Heilongjiang (HLJ, KT364261).
4	[13]	2023	North Carolina, United States	Female spayed domestic shorthair cat.	N/A	Wright Giemsa Stain	Polymerase Chain Reaction and Staining	*Colpodella* sp. (90% similarity)
5	[14]	2022	Cambodia	Dogs	N/A	NP	Next-generation sequencing (NGS)-based metabarcoding protocol	*Colpodella* sp. (95% similarity with Horse Infection #MW261750.1)
6	[15]	2023	Guiyang, China	Cats and Dogs	N/A	NP	Polymerase Chain Reaction	*Colpodella* sp. (84.71% similarity to *Colpodella* sp. ATCC 50594)
7	[16]	2023	Nakhon Si Thammarat province, Southern Thailand	Horse	*Stomoxys indicus*	NP	Polymerase Chain Reaction	*Colpodella tetrahymenae* (89.46% similarity)
8	[17]	2021	Cluj-Napoca, Romania	Human	N/A	Giemsa Stain	Morphological criteria though staining	N/A
9	[18]	2022	Ordos City, Inner Mongolia, located in northern China	Horses	N/A	NP	Polymerase Chain Reaction	*Colpodella* sp. (99.18% and 98.73% similarity with *Colpodella* sp. ATCC 50594)
10	[19]	2017	Nampula province, Mozambique	Cattle	*Rhipicephalus microplus*	NP	Polymerase Chain Reaction	*Colpodella* sp. (89% and 86% similarity)
11	[20]	2020	Warta Mouth National Park, Western Poland	Raccoon Dog (*Nycterutes procyonoides*)	N/A	NP	Polymerase Chain Reaction	*Colpodella* sp. (99.13% similarity)
12	[21]	2021	Harbin Zoo, China	Fecal Matter	N/A	NP	Polymerase Chain Reaction	*Colpodella* sp. (97% similarity with *Cryptosporidium* sp.)
13	[22]	2024	Shandong province, China	Goats and Dogs	*Haemaphysali longicornis*	NP	Polymerase Chain Reaction	*Colpodella* sp. in Dog Tick 38 (98.26% similarity with 2018 Human Infection. *Colpodella* sp. *struthionis* in Goat Tick 168 (93.66% similarity with *Cryptosporidium struthionis*) * Colpodella* sp. *yiyuansis* in Goat Tick 161 (92.98% similarity with *Colpodella tetrahymenae*
14	[23]	2020	The Greater Kafue Ecosystem, Zambia	Cattle	N/A	NP	Polymerase Chain Reaction	*Colpodella* sp. (79.6% similarity to human cases) *Colpodella* sp. (100% similarity to racoon dog case)
15	[24]	2024	Egypt	Camels	*Hyalomma dromedarii*	NP	Polymerase Chain Reaction	*Colpodella* sp. in *H. dromedarii* ticks 98.4% similarity with *Colpodella angusta*
16	[25]	2024	Italy	Cattle	*Rhipicephalus bursa*	NP	Polymerase chain reaction	100% similarity to *Colpodella* sp. strain HLJ

N/A; not applicable; NP; not performed.
